# Toward Earlier Diagnosis Using Combined eHealth Tools in Rheumatology: The Joint Pain Assessment Scoring Tool (JPAST) Project

**DOI:** 10.2196/17507

**Published:** 2020-05-15

**Authors:** Johannes Knitza, Rachel Knevel, Karim Raza, Tor Bruce, Ekaterina Eimer, Isabel Gehring, Linda Mathsson-Alm, Maryam Poorafshar, Axel J Hueber, Georg Schett, Martina Johannesson, Anca Catrina, Lars Klareskog, JPAST Group

**Affiliations:** 1 Department of Internal Medicine 3 – Rheumatology and Immunology Friedrich-Alexander University Erlangen-Nürnberg University Hospital Erlangen Erlangen Germany; 2 Department of Rheumatology Leiden University Medical Center Leiden Netherlands; 3 Division of Rheumatology, Inflammation and Immunity Brigham and Women's Hospital Harvard Medical School Boston, MA United States; 4 Department of Rheumatology Sandwell and West Birmingham Hospitals NHS Trust Birmingham United Kingdom; 5 Institute of Inflammation and Ageing University of Birmingham Birmingham United Kingdom; 6 Ocean Observations Stockholm Sweden; 7 Thermo Fisher Scientific Freiburg Germany; 8 Thermo Fisher Scientific Uppsala Sweden; 9 Division of Rheumatology Department of Medicine Karolinska Institutet, Karolinska University Hospital Stockholm Sweden

**Keywords:** rheumatology, eHealth, mHealth, symptom-checkers, apps

## Abstract

Outcomes of patients with inflammatory rheumatic diseases have significantly improved over the last three decades, mainly due to therapeutic innovations, more timely treatment, and a recognition of the need to monitor response to treatment and to titrate treatments accordingly. Diagnostic delay remains a major challenge for all stakeholders. The combination of electronic health (eHealth) and serologic and genetic markers holds great promise to improve the current management of patients with inflammatory rheumatic diseases by speeding up access to appropriate care. The Joint Pain Assessment Scoring Tool (JPAST) project, funded by the European Union (EU) European Institute of Innovation and Technology (EIT) Health program, is a unique European project aiming to enable and accelerate personalized precision medicine for early treatment in rheumatology, ultimately also enabling prevention. The aim of the project is to facilitate these goals while at the same time, reducing cost for society and patients.

## Background

The path to a correct diagnosis and efficacious treatment is often long and frustrating for patients with inflammatory rheumatic and musculoskeletal diseases (RMDs). This is a major problem as both short and long treatment efficacy depends on early and correct diagnosis. Early diagnosis for rheumatoid arthritis (RA), systemic lupus erythematosus (SLE), myositis, primary Sjögren syndrome (SS), and systemic sclerosis (SSc) is critical for improved disease outcomes and the selection of a therapy strategy. For example, in the context of RA, the first 3 months of symptoms have been identified as a therapeutic window during which immunologic mechanisms can still be altered [1,2]. Therefore, the European League Against Rheumatism (EULAR) recommends that any patient presenting with morning stiffness or joint pain or swollen joints sees a rheumatologist no later than 6 weeks after symptom onset [3]. Interestingly, the advice to see a rheumatologist for persistent joint pain poses a particular challenge, as joint pain is very common in the population [4]. However, certain variants of joint pain together with the presence of rheumatoid factor (RF) or anticitrullinated protein antibody (ACPA) indicate a high risk for the development of RA. When identified, such individuals can be given lifestyle advice to reduce the risk of disease development and an opportunity to participate in clinical trials aimed at prevention of RA. Notably, novel ways of identifying patients at risk on a large scale will be needed if ongoing preventive trials are successful and will lead to a change in clinical practice.

## Diagnostic Delay in Today’s Clinical Practice

Diagnostic delay [5] is one of the biggest current challenges in rheumatology. Rheumatic symptoms such as joint pain are common and hard to evaluate for patients and health care providers [6-8]. Patients often wait too long as they believe that the symptoms will resolve spontaneously [9] or with self-care methods [10]. General practitioners (GP) find it hard to identify RMD symptoms indicative of emerging RA or other inflammatory RMD at early stages in the disease course [11]. The resulting delay may exacerbate existing health disparities [10,11]. Furthermore, rheumatologists remain scarce worldwide [12], and this represents one of the main reasons for the delay in diagnosis. Notably, the early and correct identification of patients with emerging rheumatic diseases based solely on clinical evaluation is very challenging even for experienced rheumatologists [13].

## Improving Health Care Efficiency

**Figure 1:**
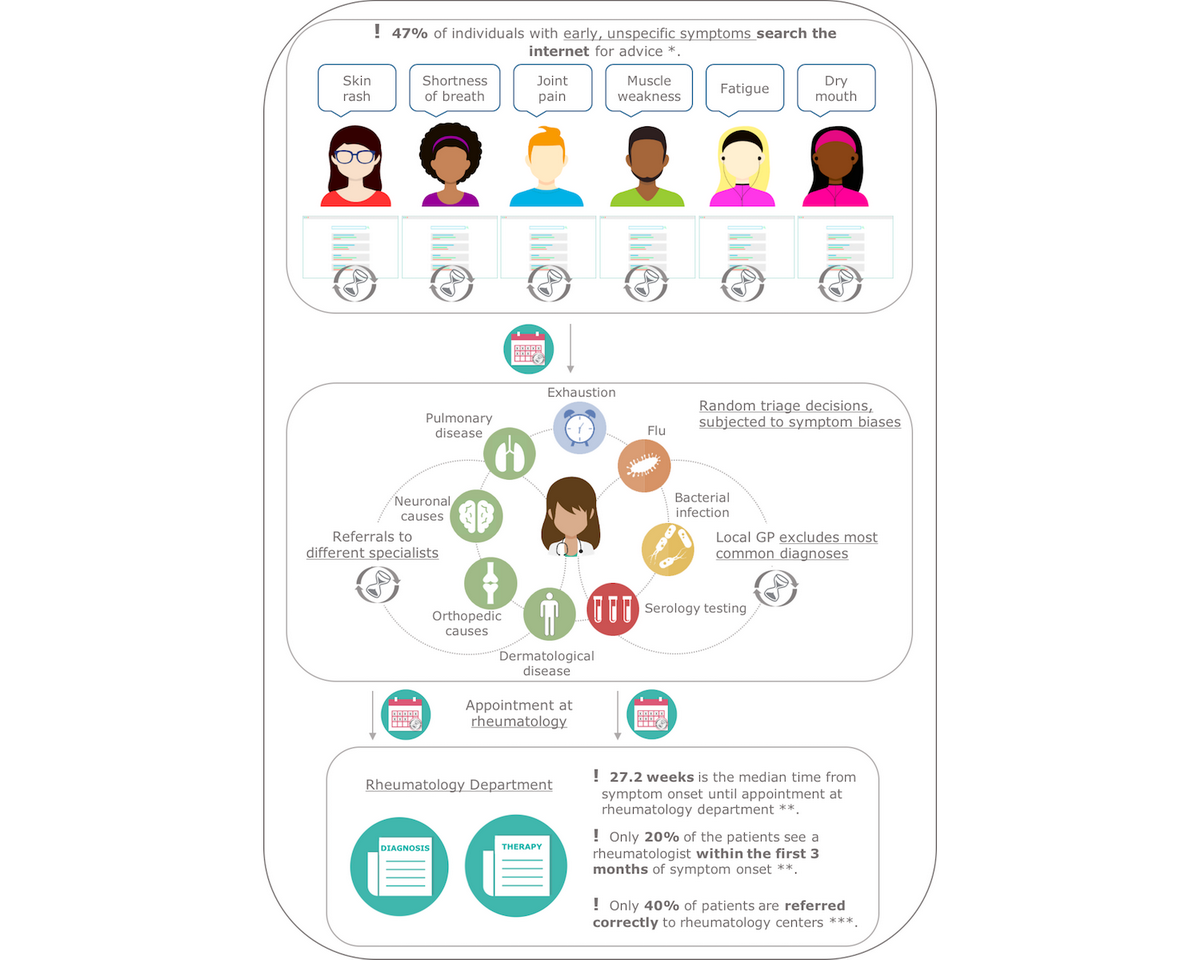
Diagnostic delay and inefficient health care service. GP: general practitioner. *Powley et al [21]. **Stack et al [10]. ***Feuchtenberger et al [15].

In addition to the challenge of reducing diagnostic delay, there is an increasing need to optimize health care efficiency. Musculoskeletal complaints account for 21.3% of the years lived with disabilities, with neck pain and low back pain accounting for almost 70% [14]. Up to 60% of patients presenting to rheumatologists in many countries turn out to have no inflammatory rheumatic diseases [15] (Figure 1). Given that the prevalence of musculoskeletal complaints increases with age, the increasing age of most populations will lead to a systemic overload of health care systems. The solution to such situations is to classically triage patients to allow prioritization based on the level of urgency and availability of effective treatment. There are different strategies [16,17] to accelerate access to rheumatologists, although low-barrier electronic health (eHealth)–based approaches remain rare. In emergency departments, heterogeneous triage decisions led to the creation and use of triage standards such as the widely used Manchester-Triage-System [18]. In rheumatology, no triage system has yet been widely accepted [16] and various local systems are being used [19]. The lack of transparent and objective standards for triage [20] represents a major hurdle for early diagnosis in patients where early treatment could make a large difference.

## The Value of Current Symptom Checkers in Rheumatology

The internet is an important source of information for both health care professionals and members of the public. Patients often check their symptoms online prior to seeing a rheumatologist [21]. Symptom checkers represent a professional alternative to search engines. They represent a patient-facing version of a diagnostic decision support system (DDSS). These systems have existed for a long time, yet are rarely used in clinical practice. Based on the patient’s reported symptoms, these systems generate a list of probable diagnoses and offer advice on further steps. These tools can potentially reduce the number of delayed and incorrect diagnoses [22]. Two systematic reviews showed that the diagnostic accuracy of physicians could be improved by using DDSS [23,24]. A retrospective evaluation that applied a DDSS on medical records of patients with rare diseases showed that the median advantage of correct disease suggestions compared to the time of clinical diagnosis was 3 months [25]. The use of symptom checkers in rheumatology has been explored only on a minor level. Powley et al [21] recently evaluated two freely available symptom checkers which included a broad variety of different diseases, and were thus not rheumatology-specific. The study showed that the help-seeking advice and diagnoses given by the symptom checkers tested was frequently inaccurate for rheumatic diseases, correctly identifying inflammatory arthritis in only 4 of 21 (19%) patients. The NHS (National Health Service) symptom checker inappropriately suggested that nearly half of patients should seek advice from emergency services. This phenomenon is well known for symptom checkers, as most systems perform in a relatively risk-adverse manner [26]. Symptom checkers promise to support patients, GPs, and rheumatologists in making the correct diagnosis in a timely manner using minimal resources with little burden; however, more evidence is needed to prove their clinical and economic benefit.

## Incorporation of Serological and Genetic Data

**Figure 2:**
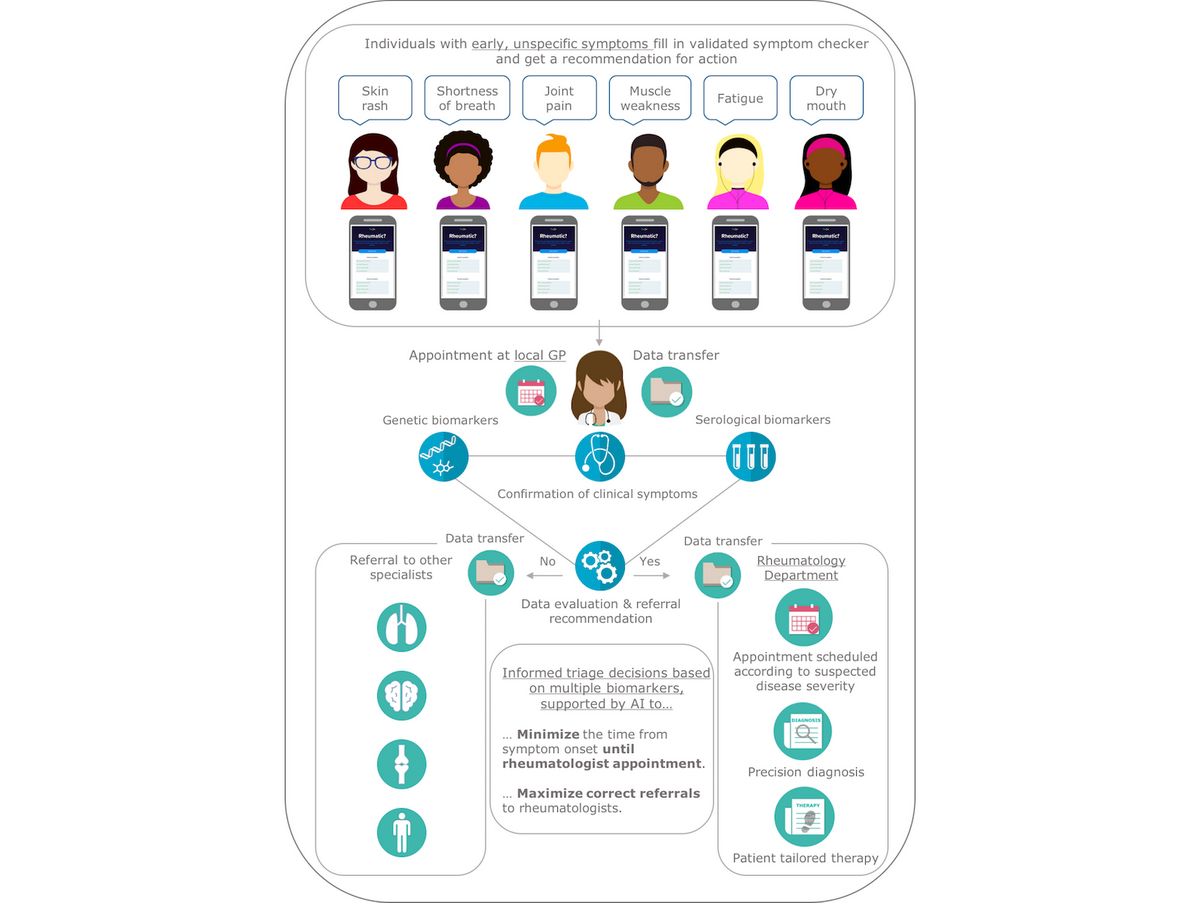
Shorter patient journey and more efficiency in health care with JPAST (Joint Pain Assessment Scoring Tool). AI: artificial intelligence; GP: general practitioner.

Subjectivity is an inherent weakness of symptom checkers. The possibility to combine the outcome from symptom checkers with serological and genetic biomarkers is likely to drastically improve early differentiation between patients with and without autoimmune inflammatory RMDs. This would shorten the time it takes for patients to get access to specialist care (Figure 2). Several serological biomarkers are included in the American College of Rheumatology (ACR)/EULAR classification criteria for RMDs (eg, ACPA and RF in RA [27], anti-dsDNA and anti-Sm in SLE [28], Scl70, anti-CENP and anti-RNA polymerase III in SSc [29], anti-Jo-1 in myositis [30] and anti-SSA/Ro in SS [31]). Such biomarkers can be present years before the onset of symptoms [32]. Early diagnostic biomarker panels and biomarkers with predictive utility are needed to guide clinical decision-making in rheumatology. The contribution of genetic variants to the individual risk of patients developing RMDs was highlighted in several twin studies [33,34]. Recently, genome-wide association studies (GWAS) have highlighted specific genes (eg, HLA, STAT4, TNF, PTPN22) for their contribution. Combinations of different genetic risk factors could contribute to the overall differentiation of patients at risk of developing inflammatory RMDs compared with other noninflammatory, nonautoimmune diseases.

Next-generation technologies have emerged, allowing fast and easy analyses of multiple serology and genetic markers. The early identification of high-risk patients allows them and their physicians to make effective lifestyle and medication changes to reduce the risk of future disease activity. The combination and interrogation of clinical, serological, and genetic data could allow the precise and early separation and description of phenotypes; currently available machine learning approaches offer particular promise in relation to this [35]. The digital documentation of symptoms and complete electronic patient records enable the identification of current treatment bottlenecks, although implementation into the digital infrastructure at the GP level and in different hospital information systems will be a challenge. Using these data, process enhancement will allow the optimization of the current health care situation [36].

## Joint Pain Assessment Scoring Tool

The objective of the Joint Pain Assessment Scoring Tool (JPAST) project, a digital diagnostic program supported by EU/EIT, is to improve the early differentiation between patients with and without autoimmune inflammatory RMDs and in addition to identify individuals at very high risk for these diseases (Figure 2). To our knowledge JPAST is the first project combining patient symptoms, genetics, and serology biomarkers for these purposes. The intention is that JPAST will eventually be tested at leading university hospitals in Europe and subsequently be implemented elsewhere to accelerate access to rheumatology services and appropriate therapy.

**Figure 3:**
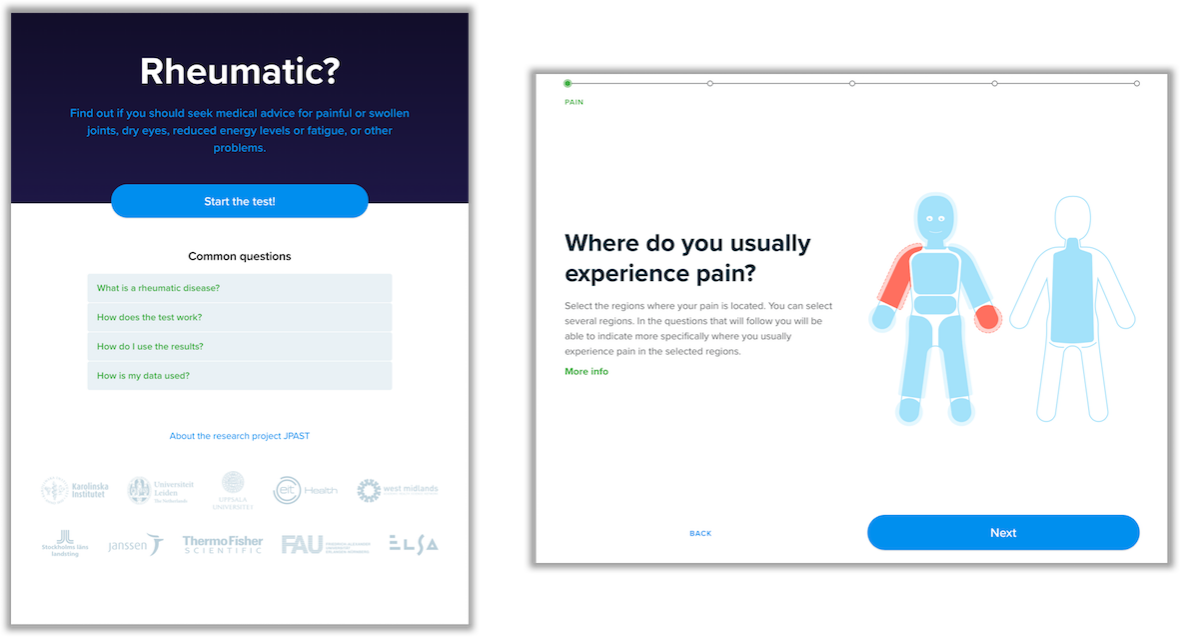
Screenshots of "Rheumatic?" the JPAST (Joint Pain Assessment Scoring Tool) symptom checker.

The current version of the JPAST eHealth tool, “Rheumatic?” (Figure 3 and Multimedia Appendix 1), is available in English, Swedish, Dutch, and German. “Rheumatic?” is a website with responsive web design, making it adaptable for smartphone and tablet use. The interface consists of single-choice buttons, multiple-choice buttons, image area questions, single-choice sliders, and pain sliders (Multimedia Appendix 1). The questions are individualized, as the next question depends on the previous answer. The scoring system was developed to be flexible and adapt to any needs that came up during the project. To achieve this, we created a system of reusable, recursive point buckets. Every point bucket has two threshold values, and a list of assigned points for any number of question options. To evaluate the point bucket, all the selected question options and their total points are calculated. The points can be positive or negative. The sum is compared to the threshold values to see if either threshold should be considered active. The recursive element comes into play when a point bucket assigns points not just directly to one particular option, but also to a threshold in another point bucket. For example, this allows us to create point buckets that are activated on individual symptoms, and then assign different scores to these symptoms for different diseases. This decouples the way points are assigned from the way the questions are asked, and allows more sophisticated scoring logic and more convenient reuse of points. Patients and physicians worked together to develop these tools.

Furthermore, specific serology-based multiplex assays and next generation sequencing panels are also being developed and will be integrated with the “Rheumatic?” eHealth tool. These assays are currently being validated using blood samples from the 5 key RMDs: rheumatoid arthritis, idiopathic inflammatory myopathies, systemic lupus erythematosus, Sjögren syndrome, and systemic sclerosis. An algorithm including genetic markers, serological markers, and clinical data will provide information to the patient and the care provider. This will help in early diagnosis of an existing disease as well as in estimating the risk for an emerging rheumatic disease in individuals with symptoms such as joint pain but without current signs of inflammation. As proposed by Weyrich et al [37] this triage system is user-friendly, dynamic, and incorporates the potential of eHealth [38]. A recent review [38] identified a lack of mHealth and eHealth tools in the field of rheumatology, underlining the innovative and stand-alone character of the JPAST project. In the first step, the diagnostic accuracy of JPAST and its usability will be analyzed in patients newly presenting to secondary care–based rheumatology clinics to test its usefulness. JPAST performance can then be compared to currently used local screening methods [17], which will allow further algorithm improvement. Once an acceptable level of performance is achieved, a prospective primary care–based long-term study should compare the clinical and economic impact of using this system versus current local care [39]. We believe JPAST will provide an accelerated pathway and improved personalized diagnosis of autoimmune inflammatory RMDs by combining innovative products and services and including all main stakeholders.

## Appendix

Multimedia Appendix 1 Screencast of "Rheumatic?" the JPAST (Joint Pain Assessment Scoring Tool) symptom checker.

## Abbreviations

ACPA: anticitrullinated protein antibody
ACR: American College of Rheumatology
DDSS: diagnostic decision support system
eHealth: electronic health
EIT: European Institute of Innovation and Technology
EU: European Union
EULAR: European League Against Rheumatism
GP: general practitioner
GWAS: genome-wide association studies
JPAST: Joint Pain Assessment Scoring Tool
NHS: National Health Service
RA: rheumatoid arthritis
RF: rheumatoid factor
RMD: rheumatic and musculoskeletal diseases
SLE: systemic lupus erythematosus
SS: Sjögren syndrome
SSc: systemic sclerosis
